# Accuracy of Electronic Health Record Documentation of Parental Presence: A Data Validation and Quality Improvement Analysis

**DOI:** 10.7759/cureus.63110

**Published:** 2024-06-25

**Authors:** Abigail Shuman, Karl Umble, Dana B McCarty

**Affiliations:** 1 Medicine, Georgetown University School of Medicine, Washington DC, USA; 2 Public Health, University of North Carolina at Chapel Hill, Chapel Hill, USA; 3 Public Health, Physical Therapy, University of North Carolina at Chapel Hill, Chapel Hill, USA

**Keywords:** parent-child relationship, accuracy of documentation, nursing documentation, neonatal intensive care unit (nicu), electronic health record (ehr)

## Abstract

Parental presence in the neonatal intensive care unit (NICU) is known to improve the health outcomes of an admitted infant. The use of the electronic health record (EHR) to analyze associations between parental presence and sociodemographic factors could provide important insights to families at greatest risk for limited presence during their infant’s NICU stay, but there is little evidence about the accuracy of nonvital clinical measures such as parental presence in these datasets. A data validation study was conducted comparing the percentage agreement of an observational log of parental presence to the EHR documentation. Overall, high accuracy values were found when combining two methods of documentation. Additional stratification using a more specific measure, each chart's complete accuracy, instead of overall accuracy, revealed that night shift documentation was more accurate than day shift documentation (76.3% accurate during night shifts, 55.2% accurate during day shifts) and that flowsheet (FS) recordings were more accurate than the free-text plan of care (POC) notes (82.4% accurate for FS, 75.1% accurate for POC notes). This research provides a preliminary look at the accuracy of EHR documentation of nonclinical factors and can serve as a methodological roadmap for other researchers who intend to use EHR data.

## Introduction

It is widely accepted that infant neurodevelopment is heavily dependent on both social and environmental exposures [1, 2]. The neonatal brain is experience-expectant, developing in a predictable, maturational sequence [3], and experience-dependent, meaning that the biological structures rely on environmental cues to activate molecular activity [4]. For all infants, but especially preterm infants cared for in the neonatal intensive care unit (NICU) [5], the relationship formed between parent and infant should begin directly after birth and is instrumental for the preterm infant’s success outside the NICU [2].

Evidence suggests that more frequent parent visitation in the NICU leads to better infant-parent attachment and, subsequently, improved parent and infant outcomes, including shorter hospitalization stays for preterm infants [6]. Researchers conclude that higher levels of visitation and maternal engagement provide opportunities for building maternal-infant attachment, which plays an important role in expediting infant recovery [6]. Early studies also showed more frequent parental visitation is correlated with an increased likelihood that an infant will be brought to their three-month follow-up appointment, a crucial appointment to ensure the development of the infant is progressing sufficiently [7]. When considering facilitators and barriers to a parent’s ability to be present at the bedside, private health insurance status, a proxy for socioeconomic status [8], is positively correlated with the likelihood of a parent being present in the NICU [9-11]. Of the commonly analyzed contributing factors, additional children in the household most significantly decrease parental presence in the NICU [9]. The physical and emotional well-being of the parent further contributes to the capacity of a parent to be present in the NICU. Traumatic birthing experiences, high levels of stress, emotional vulnerability, and feeling overwhelmed inhibit parental presence in the NICU [12-15].

Documentation of parental presence in the NICU may provide important information about families that are at greatest risk for poor health outcomes; however, because parental presence is often not considered an integral component of infant care in the NICU, there is no standard for how parental presence is documented in the medical record. Inconsistencies in documentation likely account for why few large-scale studies exist on this topic [9]. A comprehensive review in 2022 analyzed 29 studies that utilized 27 different methods of quantifying parent presence [9]. Electronic health records (EHR) are often considered the most reliable source of retrospective data from a patient’s hospitalization, including the documentation of parental presence in the NICU [9]. However, in the research community, skepticism exists about the accuracy of EHR for application in clinical research [16].

One of the greatest concerns with EHR quality is data completeness [16]. Data completeness refers to the frequency of omissions of data points for any given metric and is of particular concern when studying parental presence using a binary (present versus absent) model. The extent of data omission in EHR datasets varies drastically by health metric (e.g., acute conditions are documented more consistently than chronic conditions [16]). Data extraction methods from EHRs also influence the completeness of a dataset for use in research. Extraction errors occur most commonly with information documented in multiple places within EHRs [16,17]. One of the largest discrepancies in the accuracy of EHR data is with free-text data and its abstraction; therefore, manual review of free-text data significantly improves data completeness [16-18]. Notably, free-text data often provides crucial clinical information for both physicians and researchers retrospectively, and thus, researchers carefully consider the proper EHR extraction method for their study.

Little research exists on the validity of EHR data in the NICU setting specifically. A novel comparison paper published in 2022 compared the accuracy of maternal engagement data collection across three methods: a maternal cross-sectional survey, maternal time use diaries, and EHRs [19]. This research found that maternal exit surveys reported the highest rates of visitation, likely due to social desirability bias and errors in recollection, but data omissions were significant among all three data sets [19]. In order to ensure reliable data, several NICU-based studies have used a combination of EHR data and time logs [20,21], which complicates data collection and extraction, especially in large-scale studies [19].

The purpose of this study is to quantify the validity of the EHR dataset of parental presence recordings by nursing staff in a level IV NICU in the Southeastern US to determine if the use of EHR data is a reliable metric for investigating the effects of parental presence on infant outcomes in this NICU.

## Materials and methods

Study setting

This cross-sectional study was approved by the University of North Carolina's Institutional Review Board in November 2022 and was verified as nonhuman subject research under federal regulation (45 CFR 46.102 (e or l) and 21 CFR 56.102(c)(e)(l)).

The study was conducted in the hospital’s Level IV NICU, a 58-bed unit where approximately 750 infants are cared for annually. Nursing-to-infant ratios vary based on infant acuity, from 1:2 to 1:4.

Participants and primary data source

The hospital utilizes electronic health records through Epic® (Epic Systems Corporation, Verona, WI). In the NICU, nursing staff are trained to document parental presence in two locations within the EHR: the daily plan of care (POC) note and the infant daily cares flowsheet (FS). The POC daily note section of the chart uses a free-text format for record-keeping of all aspects of care, including parental presence and engagement. The FS uses structured data entry formatting with categorical variables. The flowsheet’s “family interaction” category includes the following fields for documenting visitors: a) mother of the child interactions; b) father of the child interactions; c) grandparent(s) interactions; and d) other visitor interactions. The frequency of input into the FS is not specified and varies based on each nurse; however, it is expected that parental presence be documented in this location at least once during the shift. The population utilized for this validation study was a convenience sample based on the infants present in the NICU during the dates of data collection. For the purposes of this study, the term parental presence was used in place of family presence despite the inclusion of grandparents in the study population to maintain consistency with analogous literature in the neonatal-perinatal field [7,9-13,20,22].

Primary data collection

This study assumed the researchers’ observations of parental presence in the NICU to be the actual observed value. Observational data were collected by two monitors in the NICU, (D.M.) and (A.S.). Two daytime shifts (12 hours each) and two nighttime shifts (12 hours each) were observed on nonconsecutive days to target different nursing staff and parent visitation patterns. The collection period of the study ran from 12/02/2022 through 12/07/2022. 

For the duration of each shift, a monitor was stationed in the lobby of the NICU with a clear view of the entrance to the unit. At the beginning of the shift, a data sheet including the bed number, patient name, and MRN was exported from Epic® into a password-protected Microsoft® Excel® spreadsheet (Microsoft Corp., Redmond, WA) to ensure patient confidentiality. When a visitor entered the unit, the monitor observed and recorded the pod and bed number to which the visitor entered. For the purposes of this research, visitation by a mother, father, or grandparent at least once at any point during the observation was considered a single parental presence visit and recorded as a (1) in the observation log. Visitor identity was verified with the head unit clerk (HUC) or nursing staff if it was not immediately clear who the family member was visiting. The HUC also acted as a monitor during short monitor breaks (five to 10 minutes). The monitor entered the information collected by the HUC into the Excel® sheet when they returned. The monitor only recorded (1) for parent visits during the shift or (0) for no parent visits during the shift. No measures of length of stay, activities during the visit, or number of family members present were recorded. Aside from the researchers and HUC, NICU staff, including nurses and physicians, all visitors were blinded to the purpose of the study to reduce the potential for bias. 

Secondary data collection

Two sources of Epic® data were collected for each observed shift in both the infant daily cares FS and the POC daily note.

The FS can be viewed in time intervals ranging from one minute to 24 hours. If no documentation was entered during the interval, the cells remain blank. During data collection, the chart was configured to show data in one-hour intervals. Documentation during the hours of the shift, including the overlapping shift change hour, was extracted from the family interaction fields. A visit (1) was recorded for any documentation (one or more entries) regarding parental presence; as mentioned above, parental presence was defined as an in-person visitation of an infant by an adult family member in order to parallel existing literature. An absence of visitors (0) was recorded with the documentation of “no contact” or phone contact only (with the staff) during the shift. If all cells were blank during the shift, (X) was recorded, indicating a complete lack of documentation, to differentiate between lack of visit and lack of documentation in our analysis. 

The POC documentation is a daily note with a free-text structure. A visit (1) was recorded only when the nurse explicitly mentioned the presence of a family member(s) in the POC summary paragraph. An absence (0) was recorded under the following circumstances: a) mention of parental contact over the phone, b) no mention of presence in person, or c) explicitly mentioned no contact with parents during the shift, or d) no mention of parental contact in the POC note. In the absence of a POC note, an (X) was recorded, again to indicate a complete lack of documentation.

Data analysis

Descriptive statistics were used to determine the overall accuracy of the data set. The data were combined into a cumulative data set for the overall percentage accuracy of recordings during the study period. The data were also stratified and analyzed by shift type (daytime vs. nighttime) and by documentation locations in Epic® (FS vs. POC note). 

After primary and secondary data were collected, the two datasets were analyzed manually by comparing the binary data on a Microsoft® Excel® spreadsheet. A success (1) was recorded when the data collected by the monitors matched the extracted data from Epic®. For each observation, accuracy was measured in four categories: overall, complete, FS, and POS. A chart was deemed to be accurate “overall” if the data in at least one of the charting locations, the POC note or the FS, matched the monitors’ log. “Complete accuracy” was designated when both the POC note and the FS documentation matched the monitors’ log. The percentage accuracy values were found by calculating the percent agreement of EHR chart data to the observed value documented by the monitor for each category.

## Results

Across all study days, the total number of opportunities for parental visitation was 193, which was found by collecting the total number of admitted infants during each shift and summing them across the four observational shifts. The NICU utilized for this study has 58 beds, and during the four shifts observed, the occupancy was 49, 47, 48, and 49 patients, respectively. Infants hospitalized over multiple days were counted in the population once for each day they were admitted. For example, if an infant was admitted for all four observational shifts, they were included four times in the overall count of 193.

Overall accuracy for nursing documentation of parental presence in the NICU, defined as accurate documentation in at least one of the charting locations (FS or POC note), was 92.2%. The percentage of charts with complete accuracy of parent presence (accurate documentation in both chart locations), was 62.7%. The FS data was significantly more accurate, with an 82.4% accuracy value, while POC documentation was accurate in 75.1% of charts (p <.05) (Table 1).

**Table 1:** Percent accuracy of nursing documentation in the EHR of parental presence *Overall accuracy is defined as accurate documentation in at least one of the charting locations. **Complete accuracy is defined as a fully accurate chart with accurate documentation in both the infant daily cares flowsheet and plan of care daily note. EHR: electronic health record

	Percent accuracy (%)
Overall accuracy*	92.2
Complete accuracy**	62.7
Charting location	
Infant daily cares flowsheet	82.4
Plan of care daily note	75.1
Shift type	
Day: overall	91.7
Day: complete	55.2
Night: overall	92.8
Night: complete	76.3

Data were also stratified based on shift type (daytime vs. nighttime). No significant difference was found in the overall accuracy of nursing documentation of parent presence on the day shift (91.67%) vs. the night shift (92.78%). However, regarding the complete accuracy of nursing documentation (both FS and POC notes matched observations), night shift documentation (76.2%) was significantly more accurate than day shift documentation (55.2%) (p < 0.05) (Table 1). 

## Discussion

This research presents an encouraging examination of nursing documentation habits in parental presence. Our results showed that the EHR may be a valid data source for parental presence measures, but individual documentation practices may hinder reliability. When combining two sources of documentation (FS and POC note), overall accuracy was high (92.2%), and the accuracy of the data improved substantially. It is important to note that the percentage of charts with complete and accurate documentation (parental presence documented accurately in both sources) is 62.7%, which is significantly lower than 92.2% for charts with accurate data in at least one area (overall accuracy). This discrepancy could present a challenge for researchers when selecting a data source from the EHR. Additional inquiry into the trends of documentation in specific units is recommended to improve accuracy. 

Our data suggest that FS data is a more accurate source of parental presence data, and thus, FS data may be more reliable than POC data. One potential explanation for this discrepancy is the usability of the FS documentation structure. The POC note is written in a free-text paragraph, whereas the FS page prompts the individual to document each metric by having a labeled row delineating locations for parental presence documentation. Furthermore, the nurses are expected to update the FS in real-time throughout the shift, while the POC note is usually completed at the end of the shift. Ample research shows that nurses working 12-hour shifts, particularly those in the NICU setting, experience fatigue, and decreased vigilance towards the end of their shifts [23,24]. The challenge with POC notes is not just for nurses to document parental presence accurately, but whether they remember to document it at all. 

Studies that use parental presence documentation from the EHR typically do not evaluate the validity of their data source and may collect data outside of the EHR, such as written parent logs and sign-in sheets [10-12,15,18]. A 2022 review published by Powers et. al. compiled a review of existing studies that quantified parental presence in some capacity [9]. Their findings showed that less than half (13/27) of the identified studies mentioned utilizing the patient’s medical record for documenting parental presence, and 19% (5/27) described parental presence as being collected in multiple documentation locations [9].

Limitations

Several limitations arose throughout this study. This study is vulnerable to human error because the analysis utilized a monitor’s observations as the true measure of parent presence. The assumption that the researcher is infallible is common in validation studies; however, it also provides a limitation to the strength of the statistic. In a larger study, using two researchers posted in different places within the NICU may improve observational accuracy. Additionally, despite a high overall number of observations (n = 193), the short observation period and single-institution structure of this research limit its generalizability to NICUs in other institutions. As previously discussed, the documentation of parental presence across health systems is not standardized, thus, any study utilizing parental presence documentation in the EHR across multiple institutions may experience inconsistent documentation practices. 

## Conclusions

The implications for this research are threefold. First, with the overall accuracy of parental presence documentation at nearly 100%, we can validate that the nursing staff’s documentation behavior aligns with the highest quality standard of care. The high level of accuracy informs internal researchers that the EHR dataset for parental presence is a valid data point for research. Using this knowledge, our team anticipates using EHR parental presence documentation as an outcome measure in future research in this specific unit. 

Second, the results provide a glimpse into the documentation of nonclinical information in the NICU. To our knowledge, no research exists analyzing the accuracy of EHR documentation of parental presence in NICU settings. This preliminary analysis provides a positive indication to disprove the skepticism surrounding the adherence to EHR documentation of nonclinical information and suggests that NICU nurses acknowledge the clinical importance of family presence for infant development.

The third implication is the use case of this methodology for data validation of future EHR datasets. If a researcher intends to utilize an EHR as the primary mode of data collection for a study, conducting a preliminary investigation into EHR accuracy may improve the strength of the findings by validating the data source. This relatively simple methodology was conducted without external funding over the course of 48 hours and provided valuable results regarding the validity of an essential outcome measure for use in future research.
